# Comparison of Aspiration Catheters with Modified Standard Catheters for Treatment of Large Pulmonary Embolism Using an In-vitro Patho-Physiological Model

**DOI:** 10.1007/s00270-021-02987-y

**Published:** 2021-11-18

**Authors:** Franziska Schubert, Masashi Tamura, Sophie Bezela, Alexander Weyers, Daniel Kütting, Matthias Menne, Ulrich Steinseifer, Johanna C. Clauser, Thomas Schmitz-Rode

**Affiliations:** 1 Department of Cardiovascular Engineering, Institute of Applied Medical Engineering, Helmholtz Institute, RWTH Aachen University and University Hospital, Aachen, Germany; 2 Department of Radiology, Keio University School of Medicine, Tokyo, Japan; 3 Institute of Applied Medical Engineering, Helmholtz Institute, RWTH Aachen University and University Hospital, Aachen, Germany; 4 Department of Radiology, University of Bonn, Bonn, Germany

**Keywords:** Pulmonary embolism, Interventional radiology, Mechanical thrombectomy, Fragmentation, Aspiration, Comparative in-vitro study, Patho-physiological model

## Abstract

**Purpose:**

The presented in-vitro study provides a comparison of various catheters for mechanical treatment of large-burden pulmonary embolism (PE) under standardized conditions, using a new test rig. Dedicated aspiration catheters (JETi®, Penumbra Indigo®, Aspirex®) were compared with standard catheters (Pigtail, Multi-Purpose, Balloon Catheter) applied for embolus fragmentation.

**Materials and Methods:**

Emboli prepared from porcine blood were washed into the test rig which consists of anatomical models of the pulmonary artery (PA) and of the right heart in combination with a pulsatile drive system. For all catheters, the duration of the recanalization procedure and the weight percentage (wt%) of the remaining, removed and washed-down clot fractions were evaluated. For aspiration catheters, the aspirated volume was measured.

**Results:**

All catheters achieved full or partial recanalization. The aspiration catheters showed a significantly (p < 0.05) lower procedure time (3:15 min ± 4:26 min) than the standard fragmentation catheters (7:19 min ± 4:40 min). The amount of thrombus removed by aspiration was significantly (p < 0.001) higher than that by fragmentation, averaging 86.1 wt% ± 15.6 wt% and 31.7 wt% ± 3.8 wt%, respectively. Nonetheless, most of the residue was fragmented into pieces of ≥ 1 mm and washed down. Only in 2 of 36 tests, a residual thrombus of 11.9 wt% ± 5.1 wt% remained in the central PA.

**Conclusion:**

Comparison under standardized in-vitro patho-physiological conditions showed that embolus fragmentation with standard catheters is clearly inferior to aspiration with dedicated catheters in the treatment of large-burden PE, but can still achieve considerable success.

**Level of Evidence:**

No level of evidence, experimental study.

## Introduction

Although a decrease in mortality related to pulmonary embolism (PE) is indicated by the WHO Mortality Database, PE causes an average of 6.5 deaths per 100,000 population annually and remains a serious clinical issue [1–3]. In particular, COVID-19 is reported to potentially promote PE resulting in sudden death even in young patients [4–6].

For patients with acute large-burden PE who cannot be treated by either systemic thrombolysis or surgical intervention, “catheter-directed therapy” is a promising approach [7–11]. It comprises mechanical thrombectomy including mechanical extraction and fragmentation of the thrombus as well as (adjunctive) catheter-mediated thrombolytic therapy [11]. Among mechanical devices, several catheters have been developed and tested over the past decades [10–12]. Standard pigtail or multi-purpose catheters have been introduced for emergency mechanical fragmentation of PE [13]. A slight modification of the pigtail catheter enabled additional rotation [14, 15]. Moreover, technically more complex solutions with aspiration were assessed for the treatment of PE, like the Aspirex® System (Straub Medical AG, Wangs, Switzerland) or the Penumbra Indigo® System (Penumbra, Inc., Alameda, CA, USA) [16, 17]. Large-bore devices that use both mechanical and aspiration mechanisms to remove the clot, such as the FlowTriever® Retrieval/Aspiration System (Inari Medical, Irvine, California), also show promising results [18]. However, the performance of such dedicated aspiration devices has not yet been compared to that of standard catheters.

The main objective of this in-vitro study is to compare dedicated aspiration catheters with slightly modified conventional catheters for fragmentation in the treatment of large PE. The term “large PE” is used in the sense of a large thrombus burden with pulmonary arterial pressure elevation. Since a valid comparison requires a standardized environment, a new test rig is proposed that provides near patho- physiological and anatomical conditions.

## Materials and Methods

### Experimental Setup

For clot preparation, porcine blood was sampled under exsanguination by cutting the carotid artery in an abattoir setting. The permission to use porcine blood was granted by the responsible veterinary authority in accordance with Article 23 of Regulation (EC) No 1069/2009. For consolidation purposes, the blood was shelved for one week in a refrigerator at 4 °C. On the day of measurement, single cylindrical clots with a length of 100 mm, a diameter of approx. 15 mm and an average weight of 6.54 g ± 0.15 g were stamped from the solidified block of clotted blood, serving in the model as thrombus with increased consistency compared to fresh thrombus.

**Fig. 1:**
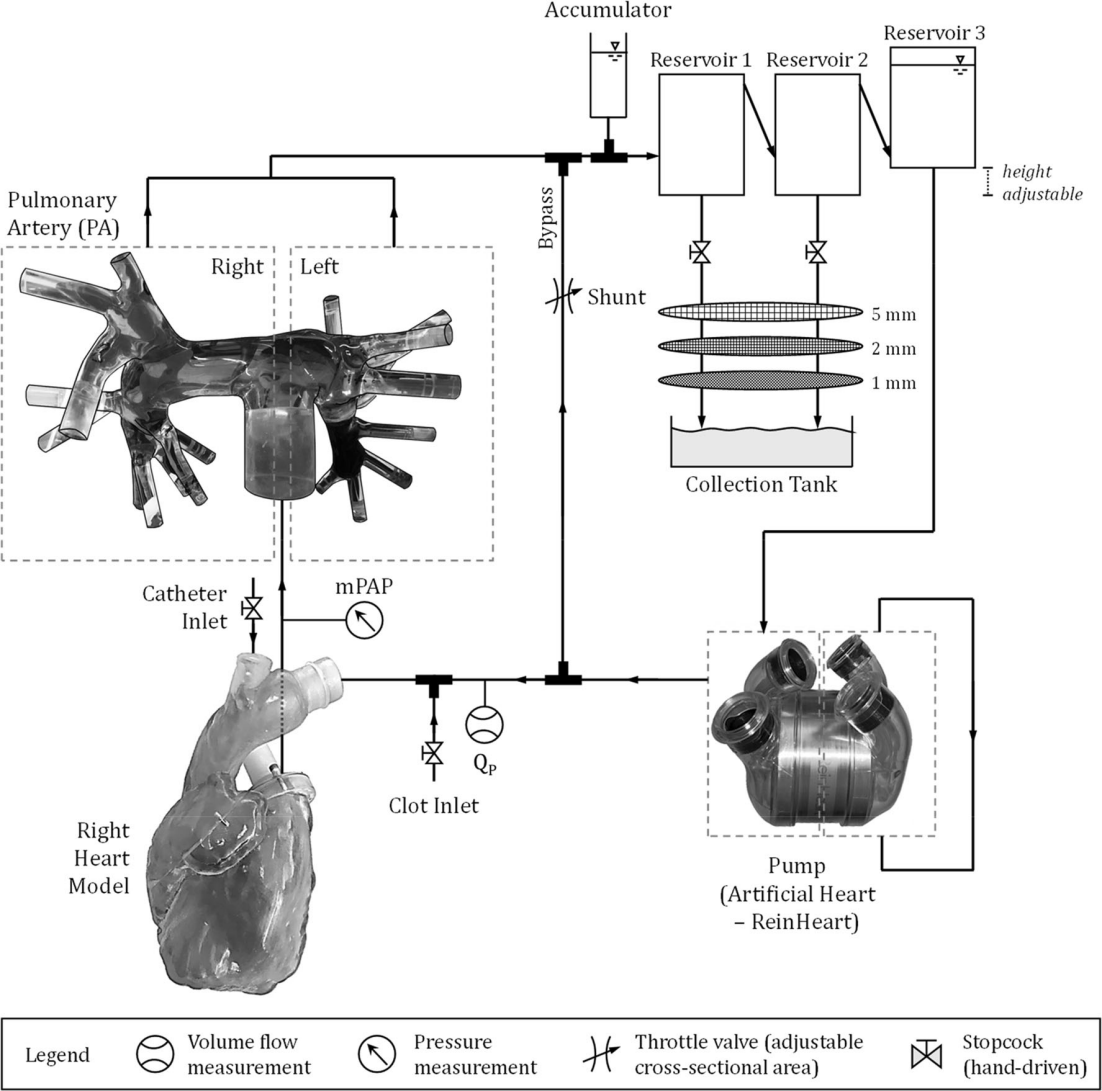
Schematic illustration of the test rig (dimensions are not representative)

A novel test rig was designed for this study to achieve anatomical and patho-physiological in-vitro conditions. The key components of the test rig (Fig. 1) are a glass model of the pulmonary artery (PA) with segmental arteries based on anatomical casts from a human corpse [19] and a transparent 3D-printed (VeroClear, Stratasys Ltd. ©, Eden Prairie, MN, USA) right heart model based on CT data. The PA model was adjusted to the right heart, mimicking the pulmonary trunk and enabling an anatomical path for the catheter. The prepared clot was washed either into the right or the left main PA. As the right heart model is rigid, the pump function had to be decoupled. Therefore, the total artificial heart ReinHeart (ReinHeart TAH GmbH, Aachen, Germany) [20] was used as a pump providing a physiological pulsatile flow of 5 L/min at 60 bpm [15]. Water at room temperature was used as fluid.

The pulmonary flow Q_P_ was measured using an ultrasound clamp-on tubing sensor (ME20PXL215/TS410, Transonic Systems Inc., Ithaca, NY, USA). The mean pulmonary artery pressure (mPAP) was monitored (Xtrans®, Codan Medizinische Geräte GmbH & Co KG, Lensahn, Germany) in the pulmonary trunk of the model. The outflow of all PA branches merged into one single tube leading to a reservoir system consisting of three closed cylindrical tank reservoirs connected in series. Clot fragments from the aspiration or fragmentation procedures were washed downstream and settled on the bottom of the first reservoir. The second reservoir functioned redundantly, catching remaining clot parts that passed the upstream reservoir. The third reservoir was attached to the pump, thus closing the loop. After the procedure, the collected clot fragments from the first two reservoirs were filtered through a set of sieves (Haver&Boecker OHG, Oelde, Germany) with filter sizes of 5 mm, 2 mm and 1 mm.

PA flow was adjusted by means of a throttle valve on a shunt bypassing the PA and the right heart model. The flow Q_P_ was set to 3.5 L/min, simulating a reduction in cardiac output caused by large thrombus burden PE.

### Experimental Interventions

**Fig. 2:**
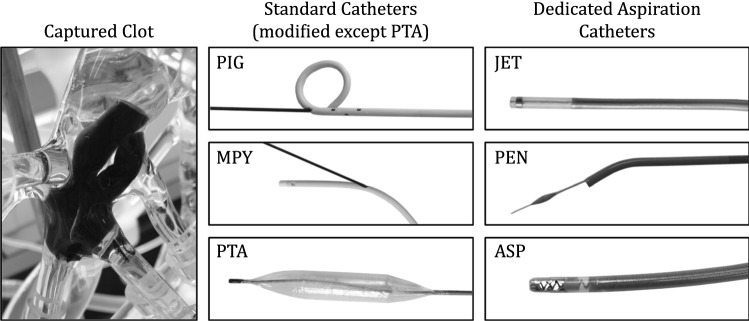
Clot captured on the right side of the pulmonary artery model (PA) (left); Investigated catheters (middle and right): PIG—Rotatable Pigtail Catheter, MPY—Rotatable Multi-Purpose Catheter, PTA—Balloon Catheter, JET—JETi® Thrombectomy System, PEN—Penumbra Indigo® System, ASP—Aspirex®

Two physicians with general experience in interventional radiology—including clinical experience in PE catheter therapy—performed all experiments. Since the test rig allowed direct sight, the procedures were conducted without the use of fluoroscopy. To mimic a jugular approach as closely as possible, the catheter was introduced at the location depicted in Fig. 1. An access sheath with a hemostasis valve was used. For placement of the catheters, similar guiding sheathes (Destination Fr. 8, Ref.: 54-86501, Terumo Medical Corp., Elkton, MD, USA) and guide wires (Radifocus®, RF-PA35153M/RF-GA35223M, Terumo; TCMTNA-35-145-3, Cook Inc., Bloomington, IN, USA) were applied unless special guide wires were provided by the catheter’s manufacturer. In Fig. 2, an example of a test embolus placed within the test setup (left) and the tested catheters (middle and right) are depicted.

### Standard Catheters for Fragmentation

With the standard catheters shown in Fig. 2 (middle row), fragmentation of the thrombus is performed either by rotation (PIG, MPY) or by repeated compression of the clot (PTA). The respective catheters and, if applicable, their modification are described below.

*PIG—Rotatable Pigtail Catheter* A commercially available high-torque 145°-angled pigtail catheter with a diameter of 6F and a length of 110 cm (Super Torque® Plus, Ref.: 533-652S, Cordis Corporation, Miami Lakes, FL, USA) was modified as proposed in previous studies [15]. An oval opening (approx. 1 × 2 mm) was cut in the outer curvature of the pigtail loop in straight projection to the catheter shaft (Fig. 2-PIG). In this way, a rotation around the axis of an inserted guide wire protruding out of the oval opening can be performed by manually rotating the proximal catheter shaft. With the pigtail tip placed in an embolus, rotation and simultaneous back-and-forth movement of the catheter achieve a fragmentation [14]. The tip of the guiding sheath was placed within the pulmonary trunk in order to reduce rotation-induced friction.

*MPY*—*Rotatable Multi-Purpose Catheter* A commercially available high-torque (Super Torque® Plus) open-end multi-purpose catheter with a diameter of 6F and a length of 100 cm (MPA2SH100cm, Ref.: 533-642, Cordis) was modified. An oval opening (approx. 1 × 2 mm) was cut in the transition between outer curvature of the deflected tip portion and the straight catheter shaft (Fig. 2-MPY). Similar to PIG, the MPY was used in conjunction with an inserted guide wire and the sheath’s tip was placed in the pulmonary trunk.

*PTA—Balloon Catheter* Placed within an embolus, repeated inflation and deflation of the balloon results in coarse fragmentation of the clot as it is pressed against the vessel’s wall. A PTA balloon catheter (Fig. 2-PTA) with a balloon diameter of 14 mm, balloon length of 40 mm, shaft diameter of 6.5F and a total length of 100 mm was selected (Zelos, Ref.: 740-1404, Optimed GmbH, Ettlingen, Germany). Based on preliminary tests with different balloon diameters and lengths, this geometry was chosen as a compromise between maneuverability and fragmentation capability.

### Dedicated Aspiration Catheters

The dedicated devices examined in this study (Fig. 2, right row) use a combination of fragmentation and aspiration to remove the thrombus.

*JET—JETi® Thrombectomy System* The JETi®-8 System (Walk Vascular, LLC., Irvine, CA, USA) with a diameter of 8F employs an interior high-pressure jet stream (saline solution) to break up the clot and to remove the crushed material (Fig. 2-JET). The jet is positioned inside the catheter lumen, enabling for clot fragmentation and simultaneous aspiration.

*PEN—Penumbra Indigo® System* The Penumbra Indigo® System (Penumbra, Inc., Alameda, CA, USA) causes mechanical clot fragmentation using a separator and subsequent suction-based extraction of the clot parts driven by a vacuum pump. In this study, a corresponding system (Fig. 2-PEN) combining an 8F aspiration catheter, a separator CAT8/SEP8 (8F, 115 cm, Ref.: CAT8XTORQ115) and an aspiration tubing (IST3) was evaluated.

*ASP—Aspirex®* The Aspirex® System (Becton Dickinson, formerly Straub Medical, Wangs, Switzerland) aspirates the clot, shreds the aspirated material and removes it from the patient’s body by suction. In the test rig, the 10F Aspirex® (S 10F, Ref.: 80231) and its special guide wire (0.018″, 4/220 cm, angled, Ref.: 80233) were examined (Fig. 2-ASP).

### Measurements

Endpoint of the study was the achievement of a complete recanalization of the central PA occlusion. The respective time was measured. In one case treatment was terminated after 20 min. Prior to each experiment, the clot was weighted. Following each procedure, reservoirs 1 and 2 were discharged and the clot residue captured in the sieves was evaluated for its weight (washed-down and filtered clot ≥ 1 mm). The fragments’ weights were summed up regarding each filter size (5 mm, 2 mm and 1 mm). Remaining clots in the PA were subsequently removed and weighted. Additionally, the weight of the removed (aspirated or fragmented) clot parts was calculated by subtracting the weight of all washed-down and remaining clot fragments from the embolus’ initial weight. For standard catheters, a washed-down clot residue with a size smaller than 1 mm, thus passing all three sieves, was considered as removed as it was no longer detectable. The respective weight percentage (wt%) referenced to the initial embolus was calculated. In the case of the aspiration catheters, the aspirated fluid volume was also measured.

### Statistical Analysis

Each catheter was tested six times (three times on each PA side). Data analysis was performed using MATLAB® (The MathWorks, Inc., Natick, MA, USA), Version R2017b. For statistical analysis, mean values and standard deviations (SD) were considered. One-way ANOVA tests were carried out using IBM® SPSS® Statistics 24 (IBM Corp. ©, Armonk, NY, USA). In the case of homogeneity of variances, post hoc tests for multiple comparison were performed using Tukey test. If variances proved inhomogeneous, Games-Howell post hoc test was applied. To assess significance between the dedicated and standard devices, single factorial ANOVA or Welch tests were performed, depending on the homogeneity of variances. Results were considered significant with p < 0.05.

## Results

**Fig. 3:**
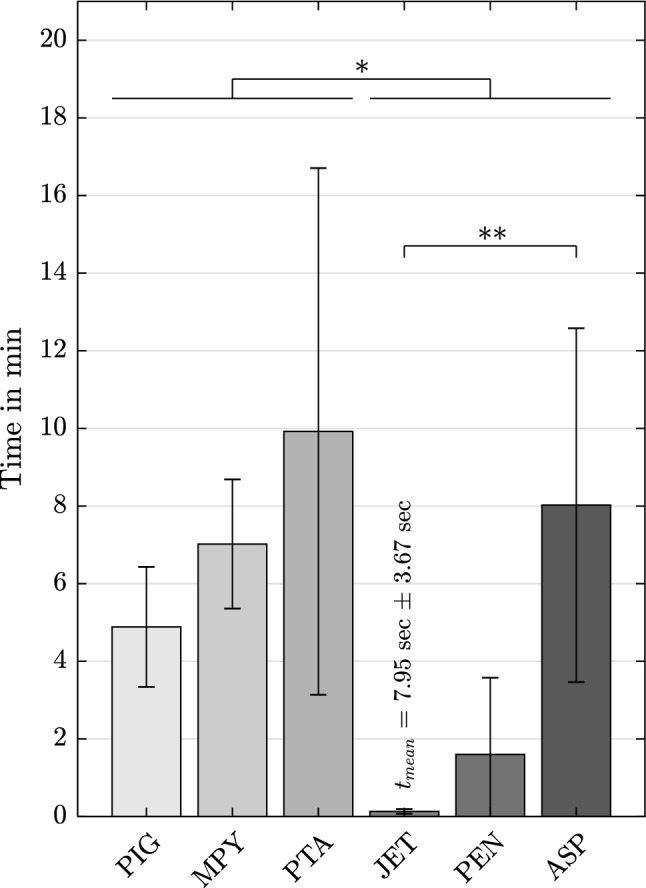
Duration of the procedure in min (mean, SD) for each catheter (n = 6, MPY: n = 4), *p < 0.05, **p < 0.005

The average time required to perform each procedure is depicted in Fig. 3. The limit of 20 min was exceeded only once using the MPY. Furthermore, one MPY catheter was damaged at the external shaft portion due to excessive torsion. Both tests are excluded from the calculation. Averaging all tests per catheter type shows a significantly lower procedure time for dedicated devices (3:15 min ± 4:26 min) compared to standard catheters (7:19 min ± 4:40 min). Notably, the most rapid thrombectomy was achieved using JET. Considering only dedicated devices, ASP led to the longest procedures with a significant (p < 0.01) deviation compared to JET. The overall maximum duration for a successful procedure was required when using the PTA device. Among standard catheters, PIG showed the shortest operation times, although no significance could be proven in comparison to the other standard devices.

**Fig. 4:**
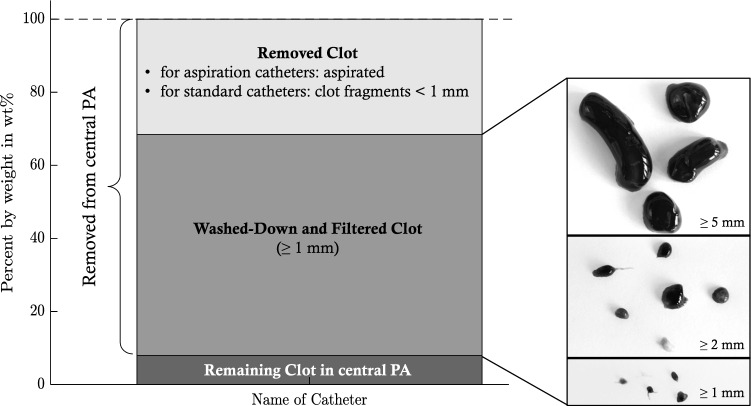
Respective fractions of the embolus in relation to its initial weight (percent by weight wt%) and example images of the individual washed-down and filtered clots divided by size; for fragmentation with standard catheters, a washed-down clot residue with a size smaller than 1 mm (thus passing all three sieves) was considered as removed as it was no longer detectable

**Fig. 5:**
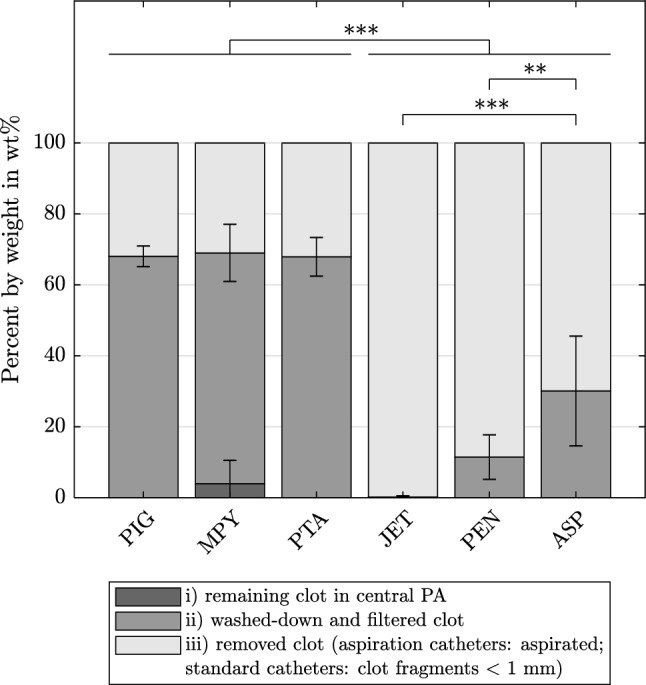
Amount of remaining clot in central PA, washed-down and filtered clot, and removed clot (aspiration catheters: aspirated; standard catheters: clot fragments < 1 mm) related to the initial clot weight in percent by weight wt% (mean, SD, per respective fraction) for each catheter, **p < 0.005, ***p < 0.001

**Fig. 6:**
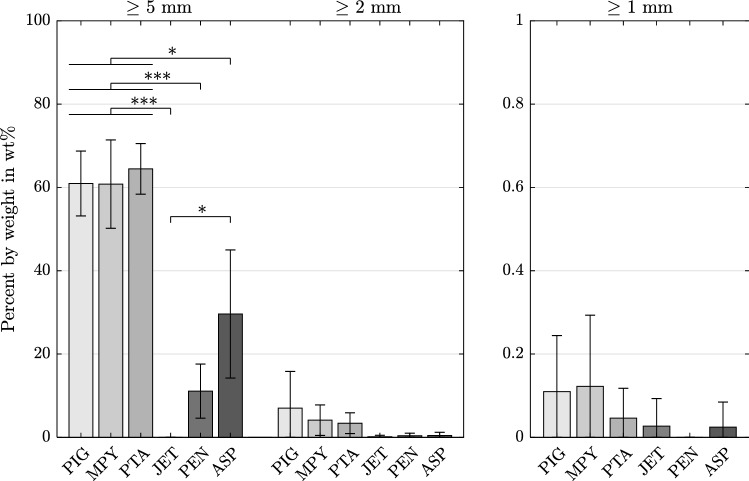
Filtered clot weights in percent by weight wt% (mean, SD) referred to the initial embolus’ weight for each catheter, summarized per clot size (≥ 5 mm, ≥ 2 mm, ≥ 1 mm), *p < 0.05, ***p < 0.001

Figure 4 explains the division into thrombus fractions after aspiration or pure fragmentation. Correspondingly, Fig. 5 presents the weight percentage (wt%) of (1) the remaining, (2) the washed-down and filtered, and (3) the removed clots. As the weight of removed clot fractions is calculated from remaining and filtered fractions, no SDs are specified within the figure. All dedicated thrombectomy devices showed a significantly (p < 0.001) higher amount of thrombus removed by aspiration (86.1 wt% ± 15.6 wt%) than by fragmentation with standard catheters (31.7 wt% ± 3.8 wt%). The JET device allowed an almost complete thrombus aspiration. The two unsuccessful trials with the MPY device led to thrombus fragments remaining in the central PA model. However, the average percentage of the corresponding residual fragments accounted for only 3.97 wt% ± 6.5 wt% of the initial thrombus (averaged over all six tests). Regarding the washed-down and filtered clot parts, the dedicated aspiration devices led to significantly (JET, PEN: p < 0.001, ASP: p < 0.05) smaller clot fractions for diameters of ≥ 5 mm than did the standard catheters (Fig. 6). However, as indicated by the high SDs, no significant differences were observed among all of the devices for clot fractions of smaller diameters (≥ 2 mm, ≥ 1 mm).

Among the dedicated aspiration devices, the ASP catheter showed a significantly higher fluid loss during aspiration (317.5 mL ± 205.5 mL), while the JET device yielded the highest fluid loss per time (452.5 mL/min ± 202.3 mL/min).

## Discussion

In scope of the presented tests, the dedicated thrombus aspiration catheters led to significantly better results in terms of the duration of the procedure and the amount of removed clots. While use of the JET device led to the most effective thrombectomy, considerably high aspiration rates were observed. In a clinical setting, lower aspiration rates might be advantageous, since serious blood loss can result if the active tip of the device is not embedded properly inside the embolus [11, 12]. Although less effective, standard catheters with a simple customization proved useful for fragmentation of large thrombus burden PE by achieving full or at least partial recanalization of the PA.

Considering their handling, PIG and MPY revealed limitations as their size (pigtail curve, deflection curvature) made fragmentation within model PA branches of smaller diameter difficult. Similar results have been reported in a clinical study [21]. Also the generation of larger thrombus fragments capable of obstructing pulmonary branches is consistent with clinical observation [14, 21].

The new test rig offers an increased level of anatomical and patho-physiological resemblance, allowing to generate PE by washing in consistent blood clots and to adjust near patho- physiological pressure and pulsatile flow values. Thus, our setup can simulate clinically observed phenomena that increase procedure time, such as difficult catheter positioning maneuvers, repositioning due to catheter dislocation, dislocation of embolus fragments, and treatment of embolus parts that moved to distal sections. The anatomical detailing of our model differs from that of published setups, for instance the investigation of the thrombectomy properties of the Straub Rotarex® Catheter (Straub Medical, Wangs, Switzerland) used a simple model with straight tubes and a straight access [22]. In the Müller-Hülsbeck model that has been used in different studies [23–25], a thrombus is cultivated inside a straight silicone tube to mimic PA embolism. With this simplified model, low intervention times have been reported for different catheters, e.g., for the Amplatz Thrombectomy Device [23], that do not correlate with clinical reports [26].

### Study Limitations

All procedures were performed under direct visual control. However, as this simplified framework applied equally to all catheters, it should not have significantly affected the comparison. As an improvement of the setup, the implementation of sight restriction in combination with x-ray fluoroscopy could be useful in future trials. Furthermore, the components of the test rig representing the PA and the right heart did not account for ventricle contraction, physiological vessel compliance or vasoconstriction. Also, the procedure-related risk of wall injury could not be evaluated. Furthermore, wall friction is artificial and may influence catheter handling. In future, it could be expedient to fabricate the anatomical structures out of elastic polymer to account for compliance. Moreover, a right heart model with a pumping function by external compression of an elastic ventricle [27] would eliminate the need for an additional pumping device. Because of the test rig’s volume (and the need to refill after each trial), water was selected as flow medium. Since pure water impairs the structural integrity of the thrombus, isotonic saline solution would be preferable. However, considering the short time frame of the tests presented, no severe impact on thrombus consistency was expected.

For future studies, blood could be considered as flow medium to include the effect of drug thrombolytic agents in the experimental setup. Additional thrombolytic therapy may be supportive in the case of fragmentation, as the thrombus surface area is increased by mechanical treatment [14, 15]. Not only blood is crucial, but also the endothelial layer of a natural vascular system and humoral factors, e.g., of an in-vivo intrinsic coagulation system, which makes it difficult to model drug thrombolysis experimentally and in-vitro.

Moreover, there was no intention of fully covering all available mechanical systems in this study. In clinical settings, large-bore thrombectomy devices with a diameter of 20-F have shown promising results [18]. The intention of our study was to create comparable framework conditions, also with regard to sheath size, which was limited to 8-F, with one exception of 10-F for the ASP catheter. A system with a catheter/sheath size of 20/22-F obviously provides better aspiration properties. However, in the clinical setting, larger system calibers may be associated with higher complication rates such as vascular injury, arrhythmia, and even ventricular fibrillation of a stressed right ventricle [17].

In our experimental setup, clot preparation was aimed at a thrombus with increased consistency and thus increased resistance to aspiration and fragmentation, although this method could not reproduce a partially organized thrombus. So far, no histological examination has been conducted to verify the exact mechanism of consolidation. The important point is that all clots were prepared according to the same protocol, so that comparability between different catheter systems is given. The need for comparability was paramount in our study, so the investigation of very different, old organized or mixed-structured clots, as found in PE in a clinical setting and certainly affecting treatability by catheter systems, was not considered.

Finally, it must be considered that regeneration after PE is a complex process that does not necessarily correlate linearly with pulmonary recanalization by catheter treatment and may be delayed. However, such a regeneration process affects treatment with all catheter systems and unfortunately cannot be reproduced in an in-vitro model.

Despite all these limitations, our setup allowed a comparison of the recanalization properties of the investigated catheters under standardized conditions. The test rig may be also applicable for training and teaching. Procedures can be performed with a direct view onto the transparent vessels or, as an improvement, under sight restriction and with the model placed in a fluoroscopy unit.

## Conclusion

In this study, a comparison of different catheters for mechanical treatment of large-burden PE was performed under standardized in-vitro conditions. It was found that although mechanical thrombectomy worked best with dedicated aspiration devices, embolus fragmentation with simple standard catheters could achieve considerable success. If dedicated PE catheters are not available in an emergency, the use of such (slightly modified) standard catheters may be a lifesaving alternative.
